# Influence of Shrub Encroachment on the Soil Microbial Community Composition of Remnant Hill Prairies

**DOI:** 10.1007/s00248-014-0369-6

**Published:** 2014-02-04

**Authors:** Anthony C. Yannarell, Sarah E. Menning, Alyssa M. Beck

**Affiliations:** Department of Natural Resources and Environmental Sciences, University of Illinois at Urbana-Champaign, 1102 S. Goodwin Ave., Urbana, IL 61801 USA

## Abstract

Hill prairies are remnant grasslands perched on the bluffs of major river valleys, and because their steep slopes make them unsuitable for traditional row crop agriculture, they have some of the lowest levels of anthropogenic disturbance of any prairie ecosystems in the Midwestern USA. However, many decades of fire suppression have allowed for shrub encroachment from the surrounding forests. While shrub encroachment of grasslands can modify soil respiration rates and nutrient storage, it is not known whether shrubs also alter the community composition of soil microorganisms. We conducted transect sampling of nine different hill prairie remnants showing varying degrees of shrub encroachment, and we used DNA-based community profiling (automated ribosomal intergenic spacer analysis) to characterize the composition of bacterial and fungal communities in the open prairie habitat, the shrub-encroached border, and the surrounding forest. While both bacterial and fungal communities showed statistically significant variation across these habitats, their predominant patterns were different. Bacterial communities of forest soils were distinct from those of the open prairie and the shrub-encroached areas, while fungal communities of the open prairie were distinct from those of the forest and the shrub-encroached border. Shrub encroachment significantly altered the community composition of soil fungal communities. Furthermore, fungal communities of heavily encroached prairie remnants more closely resembled those of the surrounding forest than those of lightly encroached prairies. Thus, shrub encroachment can cause soil fungi to shift from a “grassland” community to a “woody” community, with potential consequences for soil processes and plant-microbe interactions.

## Introduction

Hill prairies are unique prairie ecosystems on the west and southwest-facing slopes of large river valleys that can be found in the Midwestern USA [1]. In western Illinois, hill prairies maintain unique plant assemblages comprised of a mixture of prairie grasses and forbs along with sun-tolerant species from the surrounding forest, giving rise to unique plant communities that are unlike other typical tallgrass prairies [2–4]. Because of their steep slopes and dry soil, hill prairies have escaped conversion into agricultural land, and thus, they represent the Illinois prairie ecosystem with the least amount of historical human disturbance [1, 5].

Physical properties such as intense solar radiation, well-drained soil, and dry prevailing winds have caused these systems to have locally drier microclimates than the surrounding areas [6]. This inhibits forest establishment, enabling prairie flora to proliferate on the slopes even when the surrounding vegetation is predominantly forest. These systems can be conceptualized as “islands” in the midst of an otherwise forested landscape [4, 7]. Historically, these islands were likely maintained by periodic natural fires, which would prevent the establishment of woody plants [7]. However, decades of fire suppression on the landscape have contributed to the growth of forests along the river bluffs, and this has allowed for slow encroachment of trees and shrubs, resulting in the overall reduction or disappearance of hill prairies [4, 5, 8–10]. A survey of historical aerial photographs found that a majority of hill prairies in Illinois have been shrinking under encroachment of shrubs and trees from their forested margins [4]. Along the southern Illinois Mississippi River bluffs, shrub encroachment is primarily due to native smooth sumac, roughleaf dogwood, and eastern red cedar [4]. This shrub encroachment presents a threat to the unique plant assemblages of hill prairies and paves the way for accelerated forest spread [4].

Along with its overall impact on plant community composition, shrub encroachment may also impact belowground communities. Shrub encroachment negatively affects soil respiration rates, primarily through the promotion of cooler average soil conditions [11]. This indicates that shrub encroachment may be affecting overall activity rates of soil organisms. Encroachment by roughleaf dogwood and eastern red cedar has been shown to increase aboveground net primary production, decrease carbon flux from the soil, increase microbial enzyme activity, and alter net carbon storage [11–14]. Shrub encroachment has also been linked to increases in C and N mineralization rates [15–17], increases in microbial biomass C and N [18–20], and changes in the accessibility of C and N [21–23]. It is apparent that woody encroachment is having some overall effect on soil nutrient dynamics and microbial activity; however, it is unclear whether shrub encroachment also affects soil microbial community composition.

Shrubs may directly interact with soil microbes to encourage or discourage the growth of particular pathogens, parasites, commensals, and mutualists [24]. On longer time scales, shrub root exudates and litter may provide novel substrates for soil microbes, leading to succession within the soil community. All three of the most common shrubs encroaching into hill prairies (sumac, cedar, and dogwood) have been shown to have antimicrobial properties [25–27]. It is therefore reasonable to expect that encroachment by these plants may be leading to overall shifts in the community composition of soil organisms. Soil microorganisms are responsible for nutrient cycling, and microbial community composition may ultimately decide the fate of organic matter in the soil [28]. Furthermore, microbial species are agents of plant soil feedback, which can alter the outcome of plant competition and drive the process of plant community succession [29–31]. These factors can also lead to persistent soil-borne legacy effects [32, 33], and so shrub encroachment may have lasting consequences for restoration and management of threatened hill prairie ecosystems. Shifts in community composition may therefore be facilitating long-term succession from hill prairie to woodland ecosystem [4].

Here, we investigate soil microbial community composition in hill prairies along a ~60-km stretch of the Mississippi River in southwestern Illinois. We sampled hill prairies that differ in magnitude and frequency of their management activities, including burning and shrub removal, in order to determine if recent shrub encroachment alters soil microbial community composition. We hypothesize that shrub encroachment of hill prairies will change grassland soil microbial communities so that they come to more closely resemble those of the surrounding forest. We further hypothesize that this shift will be more pronounced in hill prairies with a longer history of shrub encroachment than in newly encroached prairies.

## Methods

### Study Area

The Monroe Co. hill prairie conservation corridor encompasses an approximately 60-km stretch of forested bluffs along the Mississippi River on the western border of Illinois, and this area contains numerous relict hill prairies [1, 3]. Hill prairie vegetation is predominantly composed of native prairie grasses and forbs, with little bluestem (*Schizachyrium scoparium* (Michx.) Nash), side-oats gramma (*Bouteloua curtipendula* (Michx.) Torr.), and Indian grass (*Sorghastrum nutans* (L.) Nash) being the most predominant species [4]. However, roughleaf dogwood (*Cornus drummondii* C.A. Mey.) and sumac (*Rhus* spp.) shrubs are commonly found to encroach on these prairie remnants, and some of the most heavily encroached prairies also contained honey locust (*Gleditsia triacanthos* L.) and red cedar (*Juniperus virginiana* L.) [4]. Hill prairie soils of this region belong to the Hamburg silt loam series, while those of the surrounding forest are classified as Stookey silt loam [34]. Both soil types are derived from loess, but the hill prairie soils tend to have a higher CaCO_3_ content (up to 30 % as opposed to 5 % for forest), coarser texture, shallower slope (18–35 vs. 35–70 %), and lower water holding capacity (12 vs. 21 in.) than the surrounding forest soils.

We classified prairie remnants as being subjected to light, moderate, and heavy shrub encroachment. Prairies in the light encroachment category had all been subject to shrub removal activities (cutting and or burning) within 1 year prior to sampling; as a result, they had small clusters of shrubs along their forested borders, but the central prairie “core” was free of shrubs. Prairies in the moderate encroachment category had most recently been subjected to cutting or burning 2–5 years prior to sampling. They had bands of shrubs along their forested borders, as well as prominent clusters of shrubs and/or individual shrubs within their core prairie areas. Heavily encroached prairies had not been subjected to any recent burning or shrub removal activities. Grasses and forbs in these prairies were confined to smaller patches embedded within large areas of woody vegetation. We sampled three hill prairie remnants for each of these categories of woody encroachment, for a total of nine remnants.

**Fig. 1:**
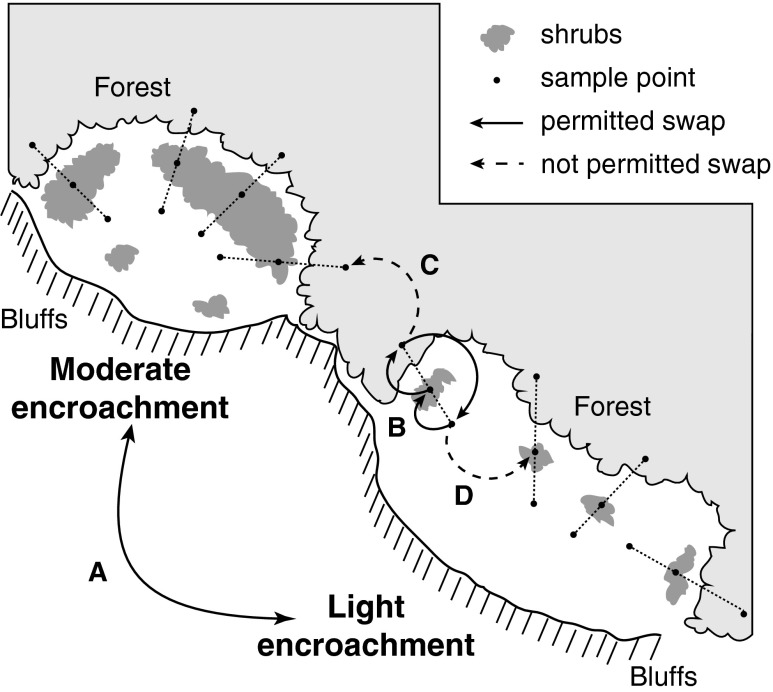
Schematic representation of the study design and analysis. The diagram shows two prairie remnants with differing degrees of shrub encroachment, as well as the surrounding forest and river bluffs. *Dotted lines* show transects and sample points spanning the three habitats (prairie, shrub, and forests). *Solid* and *dashed arrows* represent the restricted permutation scheme used to test the hypotheses about shrub encroachment level and habitat effects. For the former, we permuted encroachment level classifications across remnants (swap A). For habitat effects, we exchanged habitat levels in a serial fashion along transects (swap B), but exchanges were not permitted between transects (swaps C and D)

In each remnant, we sampled surface soils along transects (10–30 m long) spanning three different habitat types: open prairie, shrub-encroached zone, and forest (Fig. 1). After removing surface vegetation and litter, we used a handheld push probe with a 19.05-mm diameter barrel to collect the top 15 cm of soil from one prairie, one shrub, and one forest portion of each transect. All shrub samples were collected within 1 m of a dogwood or sumac stem, and in most cases, this sample was collected within a cluster of shrubs. All forest samples were collected at least 5 m from the edge of the forest canopy. Soil cores were collected into Ziploc bags and placed on ice for transport back to the laboratory. Push probe barrels were cleaned and sanitized in the field using 75 % EtOH between each core collection.

We sampled along a total of 41 transects, distributed across the nine remnants as follows: light (three, four, and four transects), moderate (four, seven, and eight transects), and heavily encroached (three, four, and four transects). At each transect, we collected three samples, one from each of the three habitat types (open prairie, shrub-encroached zone, and forest). As a result, we collected 41 samples for each habitat type, and we collected 33, 57, and 33 samples in light, moderate, and heavily encroached prairie remnants, respectively.

### Microbial Community Composition

Back in the laboratory, soils were gently homogenized inside their bags, and a subsample (approximately 20 g wet weight) of each bag was collected into a sterile 15-ml centrifuge tube, frozen immediately, and then lyophilized for 48 h. The remaining soil was air-dried in preparation for soil chemistry analyses (see below). Bulk community DNA was extracted from 0.5 g of lyophilized soil from each sample using the FastDNA SPIN kit for soil (MP Biomedicals, Solon, OH) following the manufacturer’s protocol. Extracted DNA was further purified of potential PCR inhibitors through a 15-min incubation at 65 °C with 1 % cetyl-trimethylammonium bromide and 0.7 M NaCl. Following incubations, impurities were extracted with 24:1 chloroform/isoamyl alcohol, and DNA was precipitated and washed three times with EtOH. DNA pellets were dried in a vacuum concentrator and dissolved in 1 × Tris-EDTA buffer.

Bacterial and fungal communities were characterized using automated ribosomal intergenic spacer analysis (ARISA), a length-heterogeneity PCR approach targeting the ITS region of bacterial ribosomal RNA operons and the ITS1-5.8S rRNA-ITS2 region for fungi. PCR for bacterial ARISA and fungal communities followed Yannarell and colleagues [35], using the 1406 F + 23SR primer set of Fisher and Triplett [36] for bacterial ARISA and the 2234C + 3216 T primer set of Ranjard and colleagues [37] for fungal ARISA. The 5′ ends of primers 1406 F and 3216 T were labeled with the fluorochrome dyes 6-FAM and HEX, respectively, to allow for detection of ARISA fragments during capillary electrophoresis. PCR used 20 ng of template from each sample in a final reaction volume of 25 μl, containing 5 mM Tris–HCl (pH 8.3), 0.25 mg/ml bovine serum albumin, 2.5 mM MgCl_2_, 0.25 mM of each dNTP, 0.4 μM of each primer, and 1.25 U of goTaq polymerase (Promega, Madison, WI). The following cycling conditions were used for both bacterial and fungal ARISA PCR: initial denaturation at 94 °C for 2 min, followed by 26 cycles of 94 °C for 35 s, 55 °C for 45 s, and 72 ′C for 2 min, with a final extension at 72 °C for 2 min. PCR products were diluted 1:1 with distilled H_2_O, and the resultant mixture was run on an ABI 3730XL sequencer by the W.M. Keck Center for Comparative and Functional Genomics at the University of Illinois at Urbana-Champaign.

Raw ARISA profile data were processed using the software GeneMarker (v. 1.85, SoftGenetics, LLC, State College, PA, USA) for size calling and automatic binning of peaks into operational taxonomic units (OTUs). Bins were manually corrected to remove any overlap between bins and delete bins created by spurious peaks. Peak area was used to represent the abundance of each OTU in each sample. After processing, the sample-by-OTU data matrices for bacteria and fungi were exported for statistical analysis. In addition, we recorded the total number of OTUs (peaks) in each sample as an estimate of the bacterial or fungal richness.

### Soil Chemistry

Soil pH was determined with a glass electrode after 1 h equilibration of 1 g of air-dried soil with 0.01 M CaCl_2_ [38]. KCl-extractable nitrogen was determined on an Epoch Microplate Spectrophotometer (BioTek Instruments, Winooski, VT, USA) using colorimetric development of salicylate and cyanurate reagents (Hach Co., Loveland, CO, USA) for ammonium [39] and of vanadium (III), sulfanilamide, and *N*-(1-naphthyl)-ethylenediamine dihydrochloride for nitrate [40]. Total carbon and total nitrogen content was determined by combustion of air-dried soils on an ECS 4010 CHNSO analyzer (Costech Analytical Instruments, Valencia, CA, USA).

### Statistical Analysis

Bacterial and fungal communities were analyzed separately. The rows (i.e., sample ARISA profiles) of the bacterial and fungal sample-by-OTU tables were first transformed using the Hellinger transformation [41], which standardizes the “abundance” (i.e., peak AREA) of each OTU to relative abundance through division by the total abundance of all OTUs in the sample.

Our data analysis accounted for several potential sources of nonindependence among samples arising from the study design (Fig. 1). Each transect was nested within a particular prairie remnant, and each remnant was nested within one level of the shrub encroachment treatment. There is also the potential for autocorrelated soil microbial community composition at different within- and between-remnant spatial scales. To address these statistical issues, we considered all transect level samples collected within the same habitat in the same remnant to be pseudoreplicates that did not carry a full degree of freedom for hypothesis testing. Instead, we treated our study as a split-plot design, with nine prairie remnants representing the plots (*df* = 6), encroachment level being the whole-plot factor (*df* = 2), and habitat being the sub-plot factor (*df* = 2). We used permutational multivariate analysis of variance [42] to partition the sum of squares of association matrices using the Bray-Curtis dissimilarity metric to represent “ecological distances” between samples [43]. We used the mean square ratios for a split-plot design to calculate our “pseudo-*F*” statistics, and we used a restricted permutation scheme (Fig. 1) to generate our null distributions, allowing us to account for the multiple levels of spatial dependence inherent in our study design. To test for the main effect of shrub encroachment level (whole-plot factor), we generated a null distribution by randomly swapping the levels of encroachment among the different remnants (Fig. 1, swap “A”). To test for the main effect of habitat and for the habitat-by-encroachment interaction, we restricted permutations so that samples from the same transect could only be swapped with each other (i.e., between habitat levels) in a serial fashion (Fig. 1, swap “B”). Because we used split-plot ANOVA mean square ratios, none of these tests involved the error mean square, and we were able to utilize all of our ARISA profiles without inflating our degrees of freedom.

Permutational multivariate analysis of variance was conducted in the R statistical environment [44] using the function adonis() of package vegan [45]. We generated our null distribution using 1,999 permutations of the rows of the sample-by-OTU data tables according to our restricted permutation scheme. This was accomplished by randomly shuffling blocks of rows between the nine levels of prairie, and then shuffling within each transect as a series using the shuffle() function of package permute [46]. Separate calls to adonis() were made for each permuted dataset, and the appropriate mean squares from the resulting adonis() calls were collected to calculate “pseudo-*F*” ratios for the null distribution.

To visualize patterns of community composition, we conducted nonmetric multidimensional scaling using the Bray-Curtis dissimilarity index and 100 random restarts. We also tested for homogeneity of variance within each encroachment by habitat group using the procedure of Anderson [47] with function betadisper() in package vegan.

## Results

**Fig. 2:**
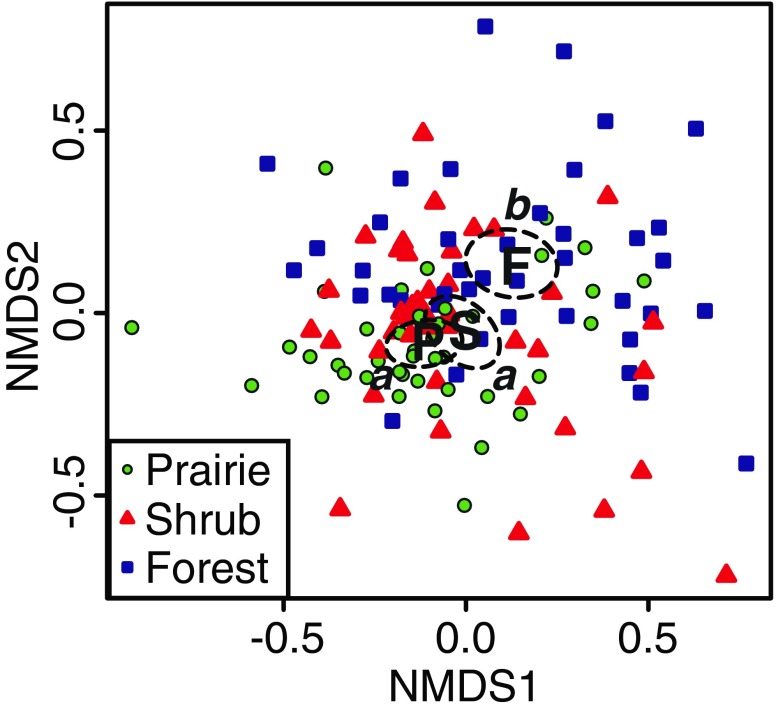
Bacterial community composition across habitats, as determined by nonmetric multidimensional scaling of Bray-Curtis community dissimilarity. *Boldface letters* provide the location of group centroids for open prairie (*P*), shrub encroached prairie (*S*), and forest (*F*) bacterial communities. *Dashed lines* display the 95 % confidence ellipses for these centroids, and the results of pairwise, post hoc comparisons are indicated by the *letters a* and *b*. NMDS1 and NMDS2 indicate the first and second ordination axes of the nonmetric multidimensional scaling solution, with a final 2-D stress of 0.23

**Fig. 3:**
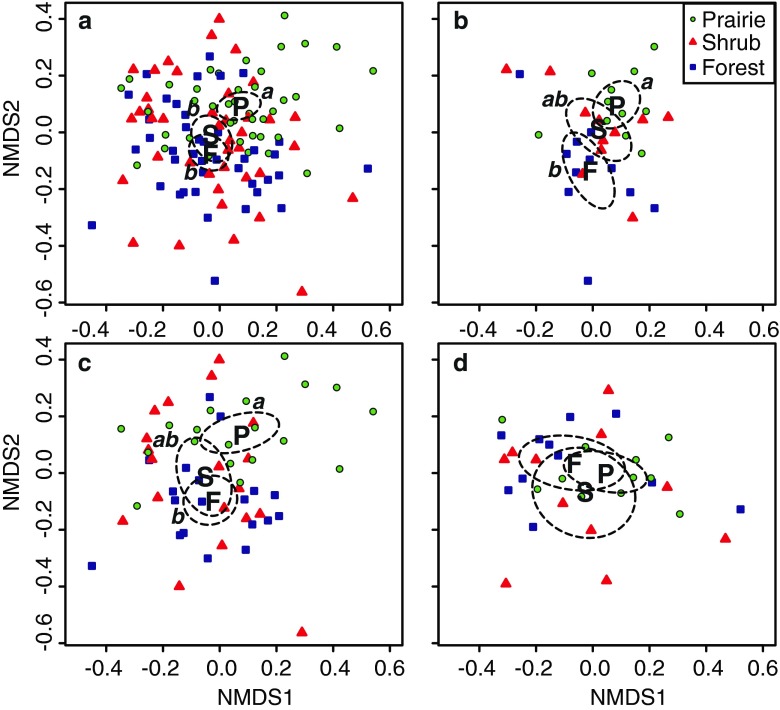
Fungal community composition across habitats, as determined by nonmetric multidimensional scaling of Bray-Curtis community dissimilarity. The interaction between habitat and encroachment level is illustrated in the different figure panels, which display the same ordination but with different subsets of the data points: **a** all data points, **b** data from lightly encroached remnants only, **c** data from moderately encroached remnants only, and **d** data from heavily encroached remnants only. *Boldface letters* provide the location of group centroids for open prairie (*P*), shrub encroached prairie (*S*), and forest (*F*) fungal communities. *Dashed lines* display the 95 % confidence ellipses for these centroids, and the results of pairwise, post hoc comparisons are indicated by the *letters a* and *b*. NMDS1 and NMDS2 indicate the first and second ordination axes of the nonmetric multidimensional scaling solution, with a final 2-D stress of 0.27

Across the entire sample set, we found significant habitat-associated variation for both bacterial (Table 1, Fig. 2) and fungal (Table 2, Fig. 3) communities. Post hoc pairwise comparisons of habitats revealed that bacterial communities from forested soils were significantly different from those of shrub-encroached areas and from grass-dominated prairie cores (*p* < 0.001 for both comparisons), but shrub-encroached bacterial communities were not significantly different from those of the prairie cores at the Bonferroni-adjusted alpha level of 0.0167 (*p* = 0.048; Fig. 2). In contrast, fungal communities were found to be significantly different in prairie cores from those of forest and shrub-encroached habitats (*p* < 0.001 for both comparisons), but forest and shrub fungal communities were not different at the Bonferroni-adjusted alpha level (*p* = 0.041; Fig. 3). Soil pH was significantly higher in prairie cores than in forest (Table 3), and there was a marginally significant trend for forest soils to have higher nitrate concentrations than core (*p* = 0.056) and shrub-encroached portions (*p* = 0.083) of prairies (Table 3). No other habitat-related soil chemical differences were detected.

**Table 1:** NP-MANOVA for bacterial community composition

Source	*df*	SS	MS	*F*	*R* ^2^	*p* value^a^
Encroachment level^b^	2	0.690	0.345	0.950	0.032	0.732
Prairie^c^	6	2.179	0.363		0.102	
Habitat^d^	2	0.981	0.491	2.868	0.046	0.001***
Encroachment by habitat	4	0.834	0.209	1.142	0.039	0.216
Prairie by habitat^e^	12	2.193	0.183		0.103	
Remainder	96	14.431			0.677	
Total	122	21.308			1.000	

^a^Tail probability of a null distribution based on 1,999 restricted permutation of samples; ***p < 0.001

^b^“Whole-plot factor” describing whether shrub encroachment in the hill prairie was light, moderate, or heavy

^c^Refers to the “plot-level” factor describing the hill prairie from which samples were collected; not to be confused with the prairie core habitat level. This MS term was needed for the denominator of the “encroachment level” test, but its significance was not tested here. *R* ^2^ is reported for comparison with other factors

^d^“Sub-plot factor” describing the position on the transect (prairie core, shrub border, forest)

^e^This MS term was needed for the denominator of the “habitat” and “encroachment by habitat” tests, but its significance was not tested here. *R* ^2^ is reported for comparison with other factors

**Table 2:** NP-MANOVA for fungal community composition

Source	*df*	SS	MS	*F*	*R* ^2^	*p* value^a^
Encroachment level^b^	2	1.248	0.624	1.058	0.027	0.385
Prairie^c^	6	3.537	0.589		0.077	
Habitat^d^	2	1.504	0.752	2.086	0.033	0.001***
Encroachment by habitat	4	1.807	0.452	1.253	0.039	0.036*
Prairie by habitat^e^	12	4.326	0.360		0.094	
Remainder	96	33.670			0.731	
Total	122	46.091			1.000	

^a^Tail probability of a null distribution based on 1,999 restricted permutation of samples; *p < 0.05, ***p < 0.001

^b^“Whole-plot factor” describing whether shrub encroachment in the hill prairie was light, moderate, or heavy

^c^Refers to the “plot-level” factor describing the hill prairie from which samples were collected; not to be confused with the prairie core habitat level. This MS term was needed for the denominator of the “encroachment level” test, but its significance was not tested here. *R* ^2^ is reported for comparison with other factors

^d^“Sub-plot factor” describing the position on the transect (prairie core, shrub border, forest)

^e^This MS term was needed for the denominator of the “habitat” and “encroachment by habitat” tests, but its significance was not tested here. *R* ^2^ is reported for comparison with other factors

**Table 3:** Chemical and biological characteristics of soils in this study

Habitat	pH	NH_4_ (ppm)	NO_3_ (ppm)	TN (%)	TC (%)	Bacteria (richness)^a^	Fungi (richness)^a^
Prairie	6.95 (0.58) a	0.26 (1.12)	2.24 (1.76) a	0.20 (0.05)	2.86 (0.76)	87.3 (25.4)	19.8 (13.2)
Shrub	6.67 (0.93) ab	0.06 (0.28)	2.31 (1.50) a	0.20 (0.05)	2.61 (0.73)	88.3 (23.0)	17.7 (14.9)
Forest	6.50 (0.91) b	1.21 (6.54)	3.23 (2.43) b	0.20 (0.08)	2.63 (1.16)	87.9 (25.6)	19.0 (15.3)

Values show the mean for each habitat type, with the standard deviation reported in parenthesis. Lower case letters in the cells indicate significant (alpha = 0.10) habitat differences based on Tukey’s honestly significant differences, and columns without letters have no significant habitat differences

^a^Richness is based on the number of OTUs present in ARISA profiles

We found a significant encroachment level by habitat interaction for fungal communities (Table 2), such that the habitat-associated differences were greatly diminished in heavily encroached hill prairies (Fig. 3b, c). There was also a significant encroachment level by habitat interaction for fungal communities in comparisons involving only prairie and forest habitats (*p* = 0.01). These habitats had distinctive fungal communities in lightly and moderately encroached hill prairies, but they were not different in heavily encroached hill prairies (Fig. 3b, c). No significant interactions were found in any tests involving bacterial communities.

**Fig. 4:**
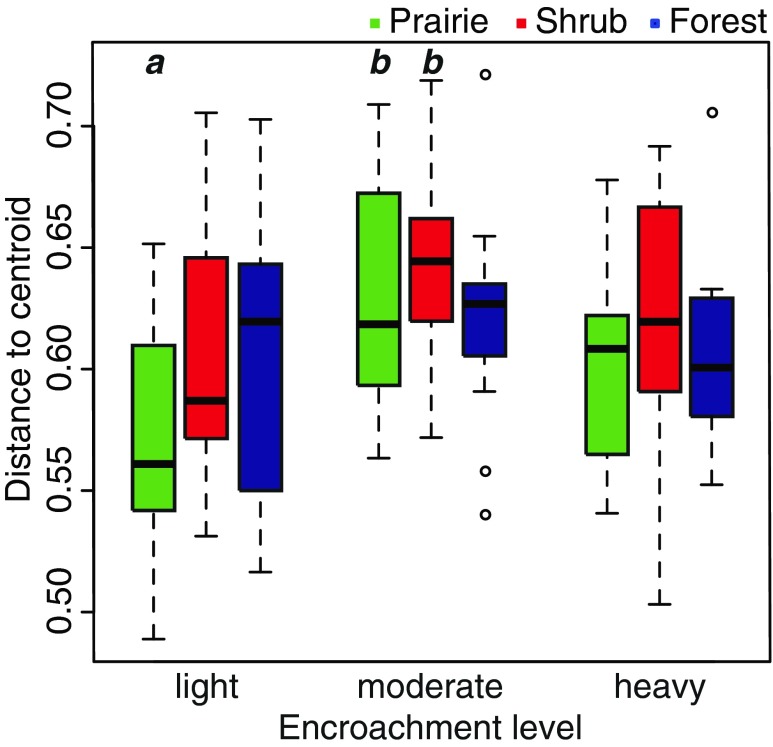
Variability of fungal community composition as determined by mean centroid distance within different habitat and shrub encroachment categories. *Boxes* show the positions of the mean (thick central line) and the first and third quartiles, and the *whiskers* extend to the most extreme data point within 1.5 “box lengths” of the mean (i.e., 1.5 × interquartile range). Categories with higher average distance to centroid (e.g., moderately encroached prairie and shrub) are more internally variable than other groups (e.g., lightly encroached prairie). The *letters above the bars* indicate treatments that were deemed to be significantly different by Tukey’s honestly significant difference post hoc comparisons; all *unlabeled bars* are considered “ab” for these comparisons

The overall level of variability of bacterial community composition was similar in each of the nine treatments groups defined by encroachment level and habitat type (*p* = 0.055). However, the variability of fungal community composition was not constant in these groups (*p* = 0.017). Post hoc comparisons using Tukey’s honestly significant differences revealed that fungal community composition in lightly encroached prairie core habitats was significantly less variable than those of moderately encroached prairie core and moderately encroached shrub habitats (Fig. 4).

## Discussion

Across the entire study area, we found that soil microbial community composition varied consistently among habitats along the prairie-to-forest continuum (Tables 1 and 2). By addressing this question in multiple hill prairie remnants and by using short transects spanning the prairie-to-forest continuum, we were able to avoid confounding the influence of habitat with the influence of large-scale spatial autocorrelation that could potentially drive microbial community differences between prairies, shrublands, and forests from different regions.

A limitation of natural experiments like ours is that we cannot definitively identify the proximate mechanisms driving microbial community change across habitats. Rather, we speculate that several nonindependent drivers of community change (beta diversity) may operate in our system. Microbial beta diversity may reflect direct and indirect effects of plants, such that plant species turnover from grass-dominated prairie to tree-dominated forest drives microbial species turnover. For example, red cedar presence in forests and heavily encroached remnants can modify soil arbuscular mycorrhizal fungal communities [48]. Grasslands and forests also differ greatly in their belowground allocation of NPP [49], and so the prairie-to-forest continuum may present soil microbes with a gradient of organic carbon available as root exudates. The litter of shrubs and grasses differ in regard to their quality and rates of decomposition [22, 48, 50], and shrub encroachment can lower fine root production and turnover rates in comparison to grasslands [50]. In addition, many of the most dominant shrubs in our hill prairies can produce antimicrobial allelochemicals [25–27], and so microbial community shifts may represent antagonistic interactions with different plant species. Finally, microbial community variability along the prairie-to-forest continuum may reflect changes in the abiotic soil environment, such as microclimate differences that have previously been documented in these systems [6]. Teasing apart the different contributions of these various factors should be an active, though challenging, area of soil microbial ecology research [51].

While the proximate drivers of microbial community change are not known, the patterns of beta diversity in our study indicate that bacteria and fungi respond to habitat change in different ways (Figs. 2 and 3). For bacteria, community composition was distinct in forest soils, while those of the open prairie and shrub-encroached prairie habitats were more similar to each other (Fig. 2). This may indicate that bacterial communities respond primarily to differences between the open habitat of the hill prairies and the closed canopy habitats of the surrounding forest. We note, however, that the forest soils in this region belong to a different soil series than the prairie soils (including the shrub-encroached portions), so we cannot discount the historical influence of soil development as playing some role in the open vs. closed habitat differences in bacterial community composition. Furthermore, forest soils had lower pH and higher available nitrate levels than prairie and shrub-encroached soils (Table 3), so these abiotic factors may act as drivers of bacterial beta diversity in addition to the potential drivers discussed above.

In contrast, we found that fungal communities were distinct in the open prairie core, with shrub and forest communities being more similar to each other (Fig. 3a). Thus, fungal communities appear to segregate between grass-dominated and wood-dominated habitats. In our study, the open prairie and the shrub-encroached portions of transects were located at the same soil series (Hamburg silt loam), with no notable differences in soil pH, N, and C (Table 3); thus, fungal community differences between prairie and shrub habitats are not confounded by the same soil factors that may affect prairie vs. forest bacteria. This result is consistent with the hypothesis that shrub encroachment alters grassland fungal community composition (Fig. 3). In light of previous works showing that shrub encroachment decreases fungal diversity [52] and alters microbial biomass [18–20] and activity [15–17], our results suggest that some of these changes may be driven by taxonomic and functional shifts in soil microbial communities. The application of high-throughput DNA sequencing and metagenomics may help elucidate the link between community structure and function in shrub-encroached soils.

### Severity of Shrub Encroachment Influences Habitat Effects

We did not detect an overall influence of shrub encroachment level on microbial community composition. However, the encroachment by habitat interaction for fungal community composition (Fig. 3) and the increased variability of fungal communities found in moderately encroached remnants (Fig. 4) suggest that the degree of shrub encroachment can influence patterns of microbial species turnover. If the encroachment of woody vegetation promotes a shift from grassland to woody fungal communities, then large contiguous areas of shrubs in heavily encroached remnants may enhance this shift. This might account for the gradual loss of habitat-specific fungal community structure seen in Fig. 3b–d.

Another impact of shrub encroachment could be increased spatial heterogeneity, leading to a corresponding increase in fungal community variation (Fig. 4). Previous studies have shown that shrublands have higher spatial heterogeneity of microbial biomass and activity [53], heterotrophic bacterial counts [54], and carbon mineralization potential [55] than grasslands. This has often been interpreted as a “resource island” effect around individual shrub plants [53–55]. We should expect these shrub island effects to be most pronounced in our moderately encroached remnants, which contained patches of shrubs in open grassland. In comparison, remnants were more homogenously grassland in lightly encroached remnants and more homogenously shrub-covered in heavily encroached remnants, with corresponding decreases in variability of fungal community composition (Fig. 4). We note that bacterial community variability showed a similar trend for higher variability in moderately encroached remnants (*p* = 0.09, data not shown), which may indicate a weakened response of soil bacteria to shrub islands.

Another possibility is that the habitat-by-encroachment level interaction for fungal community composition is driven by time lags in the relationship between the aboveground and belowground communities [33]. Because our designations of heavy, moderate, and light encroachment were related to the length of time since major shrub clearing actions (e.g., burning) had been conducted in the prairie (see “Study Area” of the “Methods” section), our shrub encroachment factor can be thought of as a coarse-scale chronosequence. From this perspective, heavily encroached prairie remnants would have a longer history of shrub encroachment and thus a longer time to develop woody fungal communities. A previous work in mesquite-encroached ecosystems has shown that larger, older shrubs have higher soil microbial biomass than younger shrubs, grass-dominated areas, and bare ground [18], suggesting that it can take some time for shrub-associated changes to microbial communities to manifest. Temporal effects of woody establishment have also been reported for microbial respiration [22] and incorporation [23] of plant-derived carbon, as well as for soil enzymatic activity, microbial activity, and microbial carbon use efficiency [12]. Because bacterial community composition showed an overall weaker relationship with shrub encroachment (Table 1, Fig. 2) than did fungi (Table 2, Fig. 3), we speculate that hill prairie fungi may be the first organisms to respond to shrub encroachment of grasslands, while bacterial changes may take longer to manifest. This interpretation is consistent with several other recent studies that indicate that fungal community composition responds more strongly to plant species changes than do bacteria [32, 35, 56].

Finally, the diminishing habitat effect in more heavily encroached remnants (Fig. 3b–d) may reflect recovery of soil fungal communities from management activities, particularly burning. Burning has been shown to reduce microbial biomass in forest soils [57, 58], and fungal biomass is more sensitive to burning than bacterial biomass in both forests and grasslands [57–59]. Because the open portions of lightly and moderately encroached remnants were recently burned prior to sampling, some of the habitat differences in these remnants may reflect the direct influence of fire. This could drive the encroachment by habitat interaction as fungal communities recover from fire over time. However, we note that fire is unlikely to explain the overall difference in fungal community composition between the open prairie and shrub habitats (Table 2, Fig. 3), as both of these areas would have been subjected to fire. Nevertheless, the influence of fire in determining microbial community dynamics should be a fruitful area of future research, especially given the historical role that fire and fire suppression have played in these hill prairie ecosystems [7].

## Conclusions

We found significant habitat-related shifts in soil microbial community structure along short transects spanning the prairie-to-forest continuum. The greatest differences in bacterial community composition were related to open vs. forested habitats, while fungal community composition differences were greatest between grassy vs. woody habitats. Shrub encroachment on hill prairies alters the soil fungal community structure, although we do not know whether these changes are brought about by direct plant-microbe interactions with shrubs (e.g., mycorrhizae) or through indirect channels mediated by litter or alterations to the soil environment. Habitat-related differences in fungal communities were diminished in more heavily encroached prairies, where shrubs have a longer time to establish without disruption from management activities, and this may indicate that time and/or shrub density are key factors related to the shift from grassland fungal communities to woody ones.
